# Effect of online aerobic exercise training in patients with bipolar depression: Protocol of a randomized clinical trial

**DOI:** 10.3389/fpsyt.2022.1011978

**Published:** 2022-11-09

**Authors:** Xueqian Wang, Huirong Luo, Yinlin Zhang, Maolin Mao, Yulin Lu, Zheng Zhang, Chunfeng Jiang, Qinghua Luo

**Affiliations:** ^1^Department of Psychiatry, First Affiliated Hospital of Chongqing Medical University, Chongqing, China; ^2^Department of Psychiatry, Banan People’s Hospital of Chongqing, Chongqing, China; ^3^Chongqing Hospital of Traditional Chinese Medicine, Chongqing, China

**Keywords:** bipolar depression, aerobic exercise, online intervention program, physical activity, pro-inflammatory cytokines

## Abstract

**Introduction:**

Bipolar disorder (BD) is a common and debilitating mental illness that affects about 400 million people worldwide, decreasing their functionality and quality of life. Medication and psychotherapy are recommended for treatment of BD, while some evidence indicates that exercise could improve the clinical outcome of BD. This study aims to investigate whether exercise intervention could reduce the mood symptoms and inflammation level of BD.

**Methods:**

This is a longitudinal, interventional, randomized, and single-blind trial. We plan to recruit 94 patients diagnosed with BD in depression episode. Patients will be randomly assigned to treatment as usual + aerobic exercise group (intervention group) and treatment as usual (TAU) only group, at a ratio of 1:1. The intervention group will undergo 40-min aerobic exercise training twice a week for eight weeks. The primary outcome of this study is the mean change of Hamilton Depression Rating Scale 17 (HAMD 17) scores from baseline to week 8. The Young Manic Rating Scale (YMRS), Self-Rating Depression Scale (SDS), and Interleukin-6 (IL-6), Tumor necrosis factor-α (TNF-α) and C-reactive protein (CRP) levels will also be measured. The measurements will be performed at baseline, immediately after intervention and two months after intervention.

**Discussion:**

Aerobic exercise training + treatment is expected to bring more benefits to BD patients than TAU only. This trial might provide stronger evidence of physical exercise efficacy for BD treatment.

**Clinical trial registration:**

This study was approved by the Chinese Clinical Trial Registry (Registration Code: ChiCTR2200057159). Registered on 1 March 2022.

## Introduction

Bipolar disorder (BD) is a chronic, recurrent, psychiatric disorder characterized by alternating episodes of depression and mania/hypomania, affecting about 2.4% of the population worldwide across diverse cultures and ethnic groups (1), leading to substantial medical burden. Serious mental illness (SMI) includes BD, major depression disorder (MDD) and schizophrenia-spectrum disorders. BD patients have high rates of suicide, disability, and low social functioning, placing a significant burden on families and society (2). BD is also the seventeenth leading cause of disability worldwide (3).

Medication and psychotherapy are commonly used to treat BD (4), but their efficacy remains unsatisfactory. Compared to manic episodes, depression episodes in BD are more incapacitating, prolonged and frequent (5). Hence, mood stabilizers, antipsychotics, and antidepressants are often used as pharmacotherapy. Lithium is commonly used and found to be effective in some patients (6), but is also more likely to cause safety events compared to placebo (7). Additionally, there is significant controversy over the use of antidepressants (8–10), which may cause mania and the treatment for mania may rebound as depressive episodes (11). Although psychosocial treatments are effective for BD (6, 12), psychotherapy is not easily accessible and expensive. Therefore, the treatment for BD remains a huge challenge, especially in depression episodes (8–10).

In this context, exercise is recommended as an adjunctive therapy for BD. Exercise has been considered useful in major depression and schizophrenia, and is recommended by the European Psychiatric Association (EPA). Exercise is considered important for people with SMI, including schizophrenia-spectrum disorders, MDD and BD, because these patients typically engage in significantly more sedentary behavior and less physical activity (13, 14), which also puts them at higher risk of developing cardiovascular and metabolic diseases (13, 15, 16). Significant evidence supports the use of exercise interventions in the treatment of MDD (17) and schizophrenia-spectrum disorders. For example, exercise could improve mental and physical heath among general population, while physical activity could reduce recurrence, relieve depressive symptoms and improve cardiorespiratory fitness and quality of life for MDD patients (18–21). The EPA suggested moderate-vigorous intensity aerobic exercise 2–3 times per week for MDD and schizophrenia-spectrum disorders, which is similar to the recommendation of the American College of Sports Medicine (ACSM) for normal population (22).

However, current evidence is insufficient to include exercise as daily recommendation for BD population since exercise-based clinical trials in this population have been consistently poor. Previous systematic reviews concluded that although exercise treatment is promising for BD, existing studies have limitations (23–26).

For example, a pilot study found that individuals with BD who participated in 40-min walking group, five times a week for 24 months reported lower depression symptoms than those who did not, without a control group (27). Another pilot study found that lifestyle intervention including exercise program showed improvement in overall functioning assessed by The Longitudinal Interval Follow-Up Evaluation-Range of Impaired Functioning Tool, but no differences in mood symptoms between groups (28). A randomized controlled trial found that 30-h aerobic exercise intervention did not augment cognition compared to the control group (29). A recent randomized trial, examining effects of N-acetylcysteine for BD, found that patients with BD who engaged more physical activity may reduce more scores on the Bipolar Depression Rating Scale. Physical activity which could enhance mitochondrial may be a novel treatment for BD (30). The possible mechanism of exercise for treating BD includes augmenting the synthesis of monoamine neurotransmitters (30–34), neurotrophin types (35, 36), and anti-inflammation. Inflammation has been shown to decrease after aerobic exercise in the general population (37) and patients with depression (38–40). Also, increasing evidence indicates that inflammatory factors are potential biomarkers of BD in evaluating severity of BD mood symptoms. Recently, a large meta-analysis indicated that C-reactive protein (CRP) and Tumor necrosis factor- α (TNF-α) might be considered as “mood episode” markers of BD, while Interleukin-6 (IL-6) was speculated to be a trait marker of BD (41). The above-mentioned data implies that CRP, TNF-α, and IL-6 could be used to examine the exercise efficacy in BD treatment.

Several researches have documented the reduction of Brain-derived Neurotrophin (BDNF) among BD patients (42, 43). At the biological level, physical exercise increases the levels of neurotransmitters, cortisol, β-endorphins, and neurotrophins such as BDNF (44, 45). BDNF could enhance neuronal ATP production in several ways (46). Also, BDNF plays an important role in brain plasticity, neuron differences and survival, neuronal function and stimulates neurogenesis by activating TrkB. There are two forms of BDNF receptors: active TrkB-FL and inactiveTrkB-T1 (47). It has been shown that physical exercise increases the levels of neurotransmitters, cortisol, β –endorphins, and neurotrophins such as BDNF, through a PGC-1α/FNDS5 pathway (48). Thus, BDNF signaling system could play a role in the pathophysiology of BD (49).

The main hypothesis of this trial is that aerobic exercise is an effective therapy to improve depressive symptoms among bipolar depression patients.

## Method and analysis

### Study objective

The primary objective of this study is to assess whether 8-week aerobic exercise will improve depressive symptoms among BD patients compared to treatment as usual. The secondary objective is to explore the effect of aerobic exercise training on inflammatory biomarkers such as CRP, IL-6, and TNF-a, manic symptoms, and cardio-respiratory fitness compared to treatment as usual. The exploratory objective is to evaluate the effects of aerobic exercise on BDNF level, emotional symptoms, quality of life, and other physical parameters, 8 weeks after intervention. The results may provide evidence for exercise therapy benefits among BD patients.

### Study setting

This trial is a longitudinal, interventional, randomized, single-blind trial to be conducted at the First Affiliated Hospital of Chongqing Medical University, China. BD patients will be randomly divided into two groups: (1) Treatment as usual + aerobic exercise group (intervention group); (2) Treatment as usual group (control group).

The treatment as usual for participants in this study would be decided by a professional psychiatrist. Patients continue to be seen for medication visit every 2–4 weeks. There is no prohibition about medication changes during the trial. All pharmacotherapy including any changes in dose or medication will be recorded in detail.

### Eligibility criteria

The participants will be recruited from the Psychiatry Outpatient Department in the First Affiliated Hospital of Chongqing Medical University, China. Research assistants will use the Mini-International Neuropsychiatric Interview (MINI), a structured diagnostic interview, to confirm the diagnosis of DSM-5 bipolar disorder depression episode before inclusion.

#### Inclusion criteria

1. Aged 18–50 years old, female or male.
2. A diagnosis of BD depression episode according to the Diagnostic and Statistical Manual of Mental Disorders-5 (DSM-5).
3. The score of HAMD ≥ 17, the score of YRMS < 6.
4. Adequate audiovisual skills to complete the necessary checks for the study.
5. Willing to participate in this trial, with signed written informed consent.

#### Exclusion criteria

1. BMI ≥ 40 kg/m^2^.
2. Systolic pressure ≥ 140 mmHg or diastolic pressure ≥ 90 mmHg.
3. Perinatal or lactation period.
4. Exercise time > 40 min per week over the last 6 months.
5. Serious physical illness (such as heart disease, diabetes, hyperthyroidism, etc.).
6. Infectious diseases, autoimmune diseases, use of anti-inflammatory drugs, cortisol hormones or antibiotics over the last 6 months.
7. Severe suicidal ideation or attempt in last 30 days or need to be hospitalized immediately due to suicidal ideation, attempt, or behaviors.

### Outcomes

The primary outcome of this study is the HAMD 17 score mean change from baseline to week 8. Participants will be assessed with HAMD 17 at baseline and week 8. The secondary outcomes are the changes of CRP, TNF-α, and IL-6 from baseline to week 8.

### Participant timeline

**FIGURE 1:**
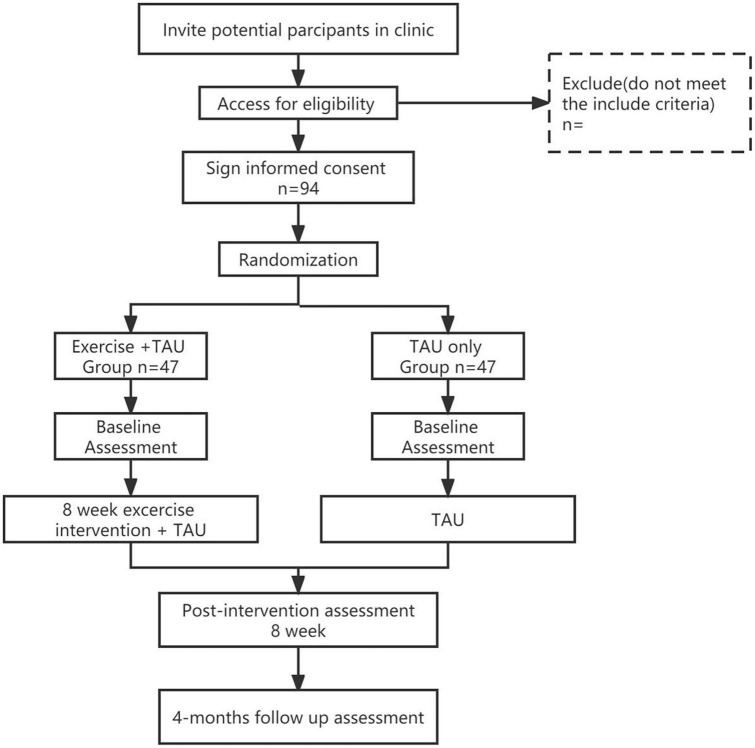
Flow of precedure. TAU, Treatment as usual.

Figure 1 shows a clear and concise timeline of the study visits, enrollment process, interventions, and assessments to be performed on participants.

### Sample size

The sample size was calculated based on the primary hypothesis. To calculate the sample size, we reviewed some literature related to the main topic and used the PASS.15 software. Power was determined as 0.09 with α = 0.05. According to related study (50), the mean value among Chinese BD patients is 13, and standard deviation is 6. It was determined that permissible error was 1/3 of the mean value, δ = 4.3, population standard deviation σ = 6. It’s necessary to have 42 samples per group. According to similar study (51, 52), it’s possible to have 20% drop-out, and we won’t replace the subjects if participants drop out. So, it is necessary to have 53 patients per group (Supplementary material).

### Recruitment

Potential participants will be recruited from the Psychiatry Outpatient Department of the First Affiliated Hospital of Chongqing Medical University, China, which is one of the largest hospitals in southwest China. A post will be put in the Psychiatry Outpatient Department to advertise this trial and patients interested in the trial can contact us for information and participation. Researchers will also call potential participants to invite them to join the study. The recruitment will be conducted from March 2022 to December 2022 (Table 1).

**TABLE 1:** Schedule of the enrollment, intervention, and assessments.

		Study period					
	
		Enrolment	Allocation	Post-allocation			
			
Time point		−t_1_	t_0_	t_1_ week 1	t_2_ week 4	t_3_ week 8	t_4_ month 4
Enrolment	Eligibility screen	×					
	Informed consent	×					
	Allocation		×				
Interventions	Exercise intervention			–	–	–	
Assessments	Sociodemographic variables		×				
	Physical parameters		×			×	×
	HMAD 17		×		×	×	×
	YMRS		×		×	×	×
	SDS		×		×	×	×
	Cardiorespiratory fitness		×			×	×
	Biological variables: CRP, TNF-α, IL-6, BDNF		×			×	×

#### Screening failures

Screening failures are defined as ineligible participants based on screening assessments. We will record the reason for screening failure, date of screening, and participant identification number.

#### Participant withdrawal or discontinuation

All participants will be free to quit the study for any reason at any time, and we will record reasons and date of dropout. Subjects will not be replaced if discontinued.

#### Allocation/Randomization

Patients will be randomized in the ratio 1:1. Random sequences will be generated by a research associate using Microsoft Office Excel 2017, and stored by hospital nurses in sequentially numbered, sealed and opaque envelopes. Researchers will open the corresponding numbered envelope after obtaining consent from each patient.

### Blinding

In this single-blinded trial, participants and main assessors will be aware of treatment allocation but not aware of the study hypothesis. Research assistants who are responsible for assessment and statistical analysis will be blinded to participant randomization assignments. Research assistants for assessment will be blinded to the treatment allocation throughout the study. An employee who is not involved in the study will enter the data into the computer so that the statistician will be unaware of the allocation.

### Assessments

#### Sociodemographic variables

Sociodemographic variables will be collected at baseline, including age, gender, ethnicity, education level, family history of mental illness, economic condition, and marital status.

#### Physical parameters

Physical parameters will be collected at baseline and immediately after 8 weeks intervention. The Ultrasonic Health Examination Scale made by Sonka Medical Co., limited (SK-X80/TCS-160D-w/h) will be used to measure the height (m) and body mass (kg). Body Mass Index (BMI) will be calculated by formula^2^: BMI = weight/height^2^.

#### Cardiorespiratory fitness

As maximum oxygen uptake (VO_2*max*_) is the gold standard of exercise physiology for evaluating cardiorespiratory fitness, Chest Step Test will be hold to assess VO_2*max*_ at baseline, with the recommendation of test manual (53).

#### Clinical variables

Individuals will be diagnosed by two psychiatrists according to DSM-5 diagnostic criteria. The Mini-International Neuropsychiatric Interview (MINI), a short structured diagnostic interview, will be used to confirm the diagnosis (54).

Depression symptoms will be measured by HMRD 17, which was designed by Hamilton in 1960 (55). The reliability of Chinese version of HMRD-17 is 0.88–0.99 and the validity is 0.92 (50). Some items are defined by increasing intensity, and others are defined by number of equal valued terms. According to Davis JM, the scores of HMRD 17 ≤ 7 indicate that individuals do not have depressive symptoms; the scores >7 and ≤17 indicate that individuals have minor or moderate depression; the score >24 indicate that individuals have major depression. Two trained psychiatrists will independently conduct the assessments. This is the most widely used clinical scale and contains 17 variables.

Depression symptoms will also be measured by the Self-Rating Depression Scale (SDS), which was designed in 1965 by Zung and recommended by the United States Department of Education, Health and Welfare in psychopharmacological research (56). SDS includes 20 items, of which 10 items are positive and 10 items are negative. The form will be directly filled by patients. The row score will be derived by adding up 20 items, with maximum possible score being 80. Index score is equal to the row score × 1.24, expressed as decimal. The standard score in China is 53 and the cutoff value of total row score is 41, which is similar to the results of a study in the USA (57).

Manic symptoms will be measured by the Young Manic Rating Scale (YMRS), which contains 11 items (58). YMRS was designed by Young in 1978, and is the most widely used manic scale worldwide. YRMS contained 11 items. Five choices are included in items 1–4, 7, 10, 11, ranging from scores 0–4, while the items 5, 6, 8, 9 have five choices scored in double points (score of 0, 2, 4, 6, 8). The score ranges from 0 to 40. When the total score ≥5 and ≤10, individuals have manic symptoms and when the score ≥22, individuals have severe manic symptoms. The reliability and validity of YRMS have been examined in a previous study.

#### Biological variables

Blood samples will be collected using EDTA anticoagulant tubes at baseline and after eight weeks of intervention, and stored at 4^°^C for less than 1 h before being sent to the laboratory at The First Affiliated Hospital of Chongqing Medical University. Plasma level of CRP will be measured by emulsion method with Roche Cobase 701. The levels of IL-6 and TNF-α will be measured by chemiluminescence with Siemens ZMM1000. *In vitro* enzyme-linked immunosorbent assay will be used to quantify the plasma of human BDNF (Table 1).

### Intervention

The intervention group will participate in online exercise program on two non-consecutive days per week for eight weeks via Tencent Conference Software. Each exercise session will include no more than eight participants. Participants will be guided and supervised by a professional coach during the entire online training. Each session will include a 10-min warm-up, a 25-min high-intensity or moderate-intensity interval training and a 10-min cool-down stretching. The training protocol is created by the first author who got fitness coach qualification certificate as well. Participants are expected to reach 70% of their age-predicted maximum heart rate (HR), which equals to 220 beats per minute-0.7 × age as an indicator of moderate intensity of aerobic exercise (59). Warm-up and cool-down exercises will include walking at low intensity (e.g., jogging in place) and stretching. Wearable devices (Huami amazfit neo smart bracelet) will be used during intervention to collect VO_2_ max.

Participants will be allowed to withdraw at any time during the trial. Some strategies will be used for adherence and compliance. For example, we will provide free follow-up service and online consulting service after program completion to encourage participants to complete the intervention. The smart bracelet will be sent to the participants for free. Completion of this program will be defined as finishing at least 50% of the exercise at each session and reaching at least 70% adherence rate of the whole intervention. The 70% adherence rate is defined as completing 11 sessions out of 16 sessions (Table 1).

### Data collection methods

Two permanent research assistants, majoring in psychology with uniform training will perform all assessments at baseline and after eight weeks of intervention using standard procedure. Blood sampling for the analysis of biological parameters will be performed during the same visit.

### Statistical analysis

SPSS 19.0 statistical software will be used for statistical analysis. Measurements will be expressed as mean ± standard deviation or median + quartiles, using *t*-test or rank sum test. Small sample composition ratios between groups will be expressed as frequency (*f*), composition ratio (P), and compared using Fisher’s exact probability method or chi-square test.

The Hamilton Depression Inventory 17-item (HAMD-17) score at eight weeks will be used as the primary outcome indicator. Differences between groups in HAMD-17 scores will be compared first by chi-square Levene test, and if the variances are chi-square, *t*-test will be used, and if the variances are not chi-square, differences within groups in HAMD-17 scores will be compared by paired *t*-test. Analysis of covariance will be used to control for baseline confounders and to further explore the differences in scores between the two groups before and after the intervention. Eight-week scores on other scales and biological indicators will be used as secondary outcomes, using similar methods as described above. Missing data will be subjected to multiple fill method, and sensitivity analysis before and after filling. An alpha = 0.05 will be taken. Intentional analysis (ITT), consistent with scenario set analysis (PP) as sensitivity analysis, will be mainly used in this study.

## Anticipated results

We anticipate that the intervention group will benefit from the aerobic exercise at the end of intervention compared to the TAU group, with less severe depressive symptoms. The intervention may increase treatment compliance of patients as well. More evidence on exercise treatment for BD is needed to prove this hypothesis. In addition to the clinical outcome, we will also investigate whether exercise could improve the quality of life and physical fitness of patients.

## Dissemination

This protocol and the study results will be shared through publication in scientific journal, and national and international conferences.

## Discussion

This randomized controlled trial is designed to investigate whether exercise therapy is effective for patients with BD. To the best of our knowledge, this is the first randomized trial investigating the direct effect of aerobic exercise in patients with BD. This trial is expected to provide current evidence for adjunct exercise therapy for BD patients with depressive symptoms.

One of the most important aims of treatment is improving the clinical outcome of patients. The treatment of BD, especially treatment of depression episode in BD, remains a major challenge. Exercise as a psychosocial adjunct for BD should be assessed with rigorous randomized clinical trials.

Many studies have been conducted to certify that aerobic exercise should be used as an adjunct therapy for depression. However, a few randomized controlled trials have been conducted to investigate whether exercise treatment is useful for BD patients. As mentioned above, there is insufficient clinical trial evidence on exercise intervention for BD. The limitations of studies on exercise intervention among BD patients included small sample sizes, no distinction between different types of exercise (lifestyle physical activity or structured intervention program), definitions of the exercise intensity, duration, and frequency of exercise. Therefore, more high-quality evidence from randomized controlled trials are essential to investigate if exercise treatment is efficacious and safe for BD. If this trial can establish that exercise treatment is beneficial for clinical outcome of BD, people suffering from this illness may achieve better clinical outcome. Aerobic exercise can be easily performed, without any major adverse events. The results of this relatively small trial may be useful for the development of future exercise regimens for BD.

This trial also has some limitation. Firstly, it is not a large sample sized trial, and the recruitment may be difficult due to some reasons, such as the 8-week intervention duration, decreased motivation, busy schedule, sedentary habits. Additionally, this trial will be supervised by professional coach and psychotherapist, which may add bias during the trial. Also, the drop out of participants may be higher than expected. To address these problems, the researchers will keep in touch with participants and their doctors. Some inexpensive gifts and free physical examination will be provided to encourage participants to finish this intervention.

## Conclusion

This is a strictly designed randomized controlled trial aimed to provide high quality evidence for adjunct treatment of bipolar disorder. Our findings may provide additional evidence for better prognosis of patients with bipolar disorder. Larger sample should be included in future studies.

## Ethics statement

The studies involving human participants were reviewed and approved by Ethics Committee of the First Affiliated Hospital of Chongqing Medical University (March 23rd, 2022, Certificate No. 2022-065). The patients/participants provided their written informed consent to participate in this study.

## Author contributions

XW and HL contributed to the conception and design of the study and wrote sections of the manuscript. YZ and CJ organized the database. ZZ, MM, and YL performed the statistical analysis. XW wrote the first draft of the manuscript. All authors contributed to manuscript revision, read, and approved the submitted version.
