# Supplementary material for: Effect of online aerobic exercise training in patients with bipolar depression: Protocol of a randomized clinical trial
Source: Front Psychiatry. 2022 Nov 9;13:1011978. doi: 10.3389/fpsyt.2022.1011978 (PMC9705266; doi:10.3389/fpsyt.2022.1011978)
Supplement: Supplementary file 1 [file Data_Sheet_1.PDF]

# Two-Sample T-Tests Assuming Equal Variance

## Numeric Results for Two-Sample T-Test Assuming Equal Variance

Alternative Hypothesis: H1: $\delta = \mu1 - \mu2 \neq 0$

| Target Power | Actual Power | N1 | N2 | N | μ1 | μ2 | δ | σ | Alpha |
|---|---|---|---|---|---|---|---|---|---|
| 0.90 | 0.90069 | 42 | 42 | 84 | 13.0 | 8.7 | 4.3 | 6.0 | 0.050 |

## References


Julious, S. A. 2010. Sample Sizes for Clinical Trials. Chapman & Hall/CRC. Boca Raton, FL.

Chow, S. C., Shao, J., and Wang, H. 2008. Sample Size Calculations in Clinical Research (Second Edition). Chapman & Hall/CRC. Boca Raton, FL.

Machin, D., Campbell, M., Fayers, P., and Pinol, A. 1997. Sample Size Tables for Clinical Studies, 2nd Edition. Blackwell Science. Malden, MA.

Zar, Jerrold H. 1984. Biostatistical Analysis (Second Edition). Prentice-Hall. Englewood Cliffs, New Jersey.


## Report Definitions

Target Power is the desired power value (or values) entered in the procedure. Power is the probability of rejecting a false null hypothesis.

Actual Power is the power obtained in this scenario. Because N1 and N2 are discrete, this value is often (slightly) larger than the target power.

N1 and N2 are the number of items sampled from each population.

N is the total sample size, N1 + N2.

μ1 and μ2 are the assumed population means.

$\delta = \mu1 - \mu2$ is the difference between population means at which power and sample size calculations are made.

σ is the assumed population standard deviation for each of the two groups.

Alpha is the probability of rejecting a true null hypothesis.

## Summary Statements

Group sample sizes of 42 and 42 achieve 90.069% power to reject the null hypothesis of equal means when the population mean difference is $\mu1 - \mu2 = 13.0 - 8.7 = 4.3$ with a standard deviation for both groups of 6.0 and with a significance level (alpha) of 0.050 using a two-sided two-sample equal-variance t-test.

## Dropout-Inflated Sample Size

| | Sample Size | | | Dropout-Inflated Enrollment Sample Size | | | Expected Number of Dropouts | | |
|---|---|---|---|---|---|---|---|---|---|
| Dropout Rate | N1 | N2 | N | N1' | N2' | N' | D1 | D2 | D |
| 20% | 42 | 42 | 84 | 53 | 53 | 106 | 11 | 11 | 22 |

## Definitions

Dropout Rate (DR) is the percentage of subjects (or items) that are expected to be lost at random during the course of the study and for whom no response data will be collected (i.e. will be treated as "missing").

N1, N2, and N are the evaluable sample sizes at which power is computed. If N1 and N2 subjects are evaluated out of the N1' and N2' subjects that are enrolled in the study, the design will achieve the stated power.

N1', N2', and N' are the number of subjects that should be enrolled in the study in order to end up with N1, N2, and N evaluable subjects, based on the assumed dropout rate. After solving for N1 and N2, N1' and N2' are calculated by inflating N1 and N2 using the formulas N1' = N1 / (1 - DR) and N2' = N2 / (1 - DR), with N1' and N2' always rounded up. (See Julious, S.A. (2010) pages 52-53, or Chow, S.C., Shao, J., and Wang, H. (2008) pages 39-40.)

D1, D2, and D are the expected number of dropouts. D1 = N1' - N1, D2 = N2' - N2, and D = D1 + D2.

# Two-Sample T-Tests Assuming Equal Variance

## Procedure Input Settings

### Autosaved Template File
C:\Users\ASUS\Documents\PASS 15\Procedure Templates\Autosave\Two-Sample T-Tests Assuming Equal Variance - Autosaved 2021_12_10-19_14_4.t388

### Design Tab

| | |
|---|---|
| Solve For: | Sample Size |
| Alternative Hypothesis: | Two-Sided |
| Power: | 0.9 |
| Alpha: | 0.05 |
| Group Allocation: | Equal (N1 = N2) |
| Input Type: | Means |
| $\mu 1$: | 13 |
| $\mu 2$: | 8.7 |
| $\sigma$: | 6 |
